# Identifying Chagas disease vectors using elliptic Fourier descriptors of body contour: a case for the cryptic *dimidiata* complex

**DOI:** 10.1186/s13071-020-04202-2

**Published:** 2020-07-01

**Authors:** Daryl D. Cruz, Elizabeth Arellano, Dennis Denis Ávila, Carlos N. Ibarra-Cerdeña

**Affiliations:** 1 Centro de Investigación en Biodiversidad y Conservación (CIByC), UAEM, Cuernavaca, Morelos México; 2 Departamento de Biología Animal y Humana, Facultad de Biología, Universidad de La Habana, Havana, Cuba; 3 Departamento de Ecología Humana, Centro de Investigación y de Estudios Avanzados del IPN (CINVESTAV), Unidad Mérida, Yucatán, México

**Keywords:** Triatomine, Identification, Morphometric analysis, Contours, Fourier

## Abstract

**Background:**

*Triatoma dimidiata* (Reduviidae: Triatominae) is an important vector of Chagas disease in various countries in the Americas. Phylogenetic studies have defined three lineages in Mexico and part of Central America. While there is a marked genetic differentiation, methods for identifying them using morphometric analyses with landmarks have not yet been fully resolutive. Elliptical Fourier descriptors (EFDs), which mathematically describe the shape of any closed two-dimensional contours, could be a potentially useful alternative method. Our objective was to validate the use of EFDs for the identification of three lineages of this species complex.

**Method:**

A total of 84 dorsal view images of individuals of the three lineages were used. Body contours were described with EFDs using between 5 and 30 harmonics. The number of obtained coefficients was reduced by a principal components analysis and the first axis scores were used as shape variables. A linear discriminant function analysis and an ordination plot of the discriminant analysis were performed using the shape variables. A confusion matrix of the ordination plot of the discriminant analysis was obtained to estimate the classification errors, the first five PC scores were statistically compared, and a neural network were then performed using the shape variables.

**Results:**

The first principal component explained 50% of the variability, regardless the number of harmonics used. The results of discriminant analysis get improved by increasing the number of harmonics and components considered. With 25 harmonics and 30 components, the identification of haplogroups was achieved with an overall efficiency greater than 97%. The ordering diagram showed the correct discrimination of haplogroups, with only one error of discrimination corroborated by the confusion matrix. When comparing the first five PC scores, significant differences were found among at least two haplogroups. The 30 multilayer perceptron neural networks were also efficient in identification, reaching 91% efficiency with the validation data.

**Conclusions:**

The use of EFD is a simple and useful method for the identification of the main lineages of *Triatoma dimidiata*, with high values of correct identification.
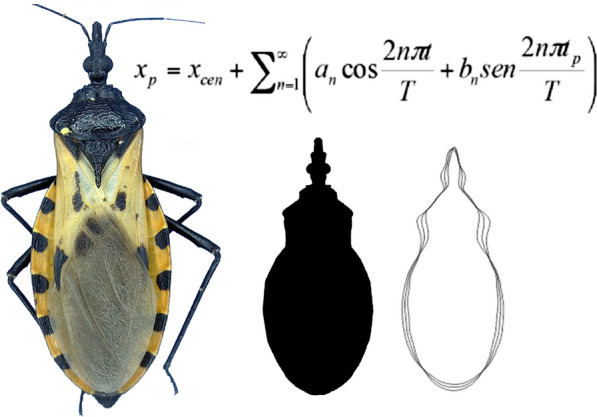

## Background

Cryptic species are one of the great challenges for systematic biologists since, in many cases, speciation is not accompanied by distinctive morphological characters and allopatric distributions that facilitate the identification of different entities at the species level [1, 2]. For that reason, the actual number of biological species is likely to be greater than the current nominal species count, most of which are delineated by purely morphological characteristics.

Research focused on cryptic species has increased over the last two decades mainly by the availability of DNA sequences [2]. The use of the term has grown and refers to two or more distinct species that are erroneously classified (hidden) under a single taxonomic entity, but through other evidence, mainly genetic, it can be proved that they have followed different evolutionary paths [3]. Cryptic species are found in almost all groups of organisms, and in the case of insects, their presence is a very frequent phenomenon in several orders [4, 5]. In the field of epidemiology, the correct identification of species in insect groups with medical importance is a key component for the design of vector control and surveillance strategies [6]. This is mainly because different species may vary in terms of their competence as vectors and their epidemiological importance as well as in their susceptibility to insecticides or other control strategies [7].

One of the most epidemiologically important groups of insects on the American continent is the triatomines (Triatominae: Reduviidae), the vectors of Chagas disease (CD). In this group, the genus *Triatoma* is the most diverse genus [8]; approximately 70 species have been described and it is the genus with the largest geographical distribution within the subfamily [9, 10]. Multiple inter- and intraspecies taxonomic questions have arisen in this group, with species repeatedly included and excluded from different complexes throughout the history of the study of their systematics and taxonomy [11–14]. The combination of unresolved taxonomic relationships and the detection of cryptic species within this genus highlight the need to address the systematics of this group [15–18]. The phenomenon of cryptic speciation is common in the Triatominae [19–21] and results in species that are nearly identical morphologically, which often makes identification based only on traditional morphological characters difficult or impossible.

The identification of triatomine species has usually been carried out using traditional morphometry [11, 14, 22, 23]. However, the use of geometric morphometry has led to new techniques for evaluating morphological characters in a taxonomic context; it complements the use of other methods of discrimination [24] and has been used for the recognition of very close species with a long history of controversy among taxonomists [25] and apparently cryptic species, including some of the genus *Triatoma* [7, 26–32].

The *Triatoma dimidiata* complex represents one of the major vectors of Chagas disease in all the countries where it is distributed [18, 33, 34]. It is present in Mexico, all the countries of Central America, Colombia, Ecuador and Perú [18, 35]. Throughout its range, it can be found in jungle, peridomestic and domestic habitats, where non-domiciled populations act as sources of re-infestation and participate in the transmission of the parasite to humans [35–38].

Phylogenetic studies using sequences from *cytb*, *nad4*, and *16S* rRNA genes, have defined three lineages in Mexico and part of Central America (with 6–14% divergence among haplogroups) [15, 39], which were recently reaffirmed by Pech-May et al. [18]. Using geometric morphometry techniques with a landmark-based analysis, Gurgel-Gonçalves et al. [40] reached correct identification rates of 70.5%, 76.7% and 82.5% for haplogroups 1, 2 and 3 of *T. dimidiata* respectively. More recently, Khalighifar et al. [41] using TensorFlow [42], an open-source software platform, representing the most recent addition to the deep learning toolbox [43] (Google Brain Team; https://research.google.com/teams/brain/), were able to increase the correct classification of specimens of the three haplogroups (84.1% H1, 86.7% H2 and 87.5% H3) [41]. Although these methodologies are the cutting-edge approach to the automatized species identification within the Triatomine group, this rate of identification is still insufficient and methods that guarantee higher power of correct discrimination are still necessary.

As an alternative, in this study we propose the use of elliptical Fourier descriptors (EFDs), which can delineate any shape with a two-dimensional closed contour, as suggested by Kuhl and Giardina [44]. Contour analysis is based on the digitalization of the silhouette of an object, which is expressed as a sequence of coordinates (x, y) that can be manipulated mathematically and adjusted to an equation derived from Fourier functions. For the extraction and digitization of outline characters, the elliptic Fourier algorithm has the advantages of being able to reconstruct outlines, eliminate errors in orientation caused by interference, size images and trace the starting point of an original image [45–48]. This method has been widely applied to the analysis of various biological shapes [48, 49] and more recently, as a tool for pattern detection, correct insect identification and automatic identification systems [50–52]. For the Triatominae in particular, the elliptic Fourier algorithm has been used with the objective of identifying species from the analysis of different structures [27, 53].

Here, we apply EFDs in order to evaluate their ability to identify the three described *T. dimidiata* haplogroups for Mexico and part of Central America. The results of this evaluation contribute to the implementation of tools for accurate discrimination between triatomine species and potentially to the control and prevention of CD.

## Methods

### Sample information

In order to test the ability of EFDs to discriminate among the haplogroups of *T. dimidiata*, we used the images obtained by Gurgel-Gonçalves et al. [40], which are available in the Dryad Digital Repository (http://dx.doi.org/10.5061/dryad.br14k). The original series of photos for triatomines were taken from entomological collections across Mexico (Centro Regional de Investigación en Salud, Instituto Nacional de Salud Pública, México; Laboratorio Estatal de Salud Pública de Guanajuato; Universidad Autónoma Benito Juárez, Oaxaca; Universidad Autónoma de Nuevo León, Monterrey), and 44, 30 and 40 images of haplogroups 1, 2 and 3 were obtained, respectively, with which the automated identification process tested by Gurgel-Gonçalves et al. [40] was performed. For this study, only images that had the necessary characteristics to perform the contour analysis were selected, i.e. only images with an unmodified contour and wings that were not broken or overlapped. This filtering process resulted in a total sample of 37 (21♀, 16♂), 23 (17♀, 6♂) and 36 (17♀, 19♂) images for haplogroups 1, 2 and 3, respectively. The conditions under which the photographs were taken, and more information about the samples, are detailed in Gurgel-Gonçalves et al. [40].

### Images manual pre-processing

**Fig. 1:**
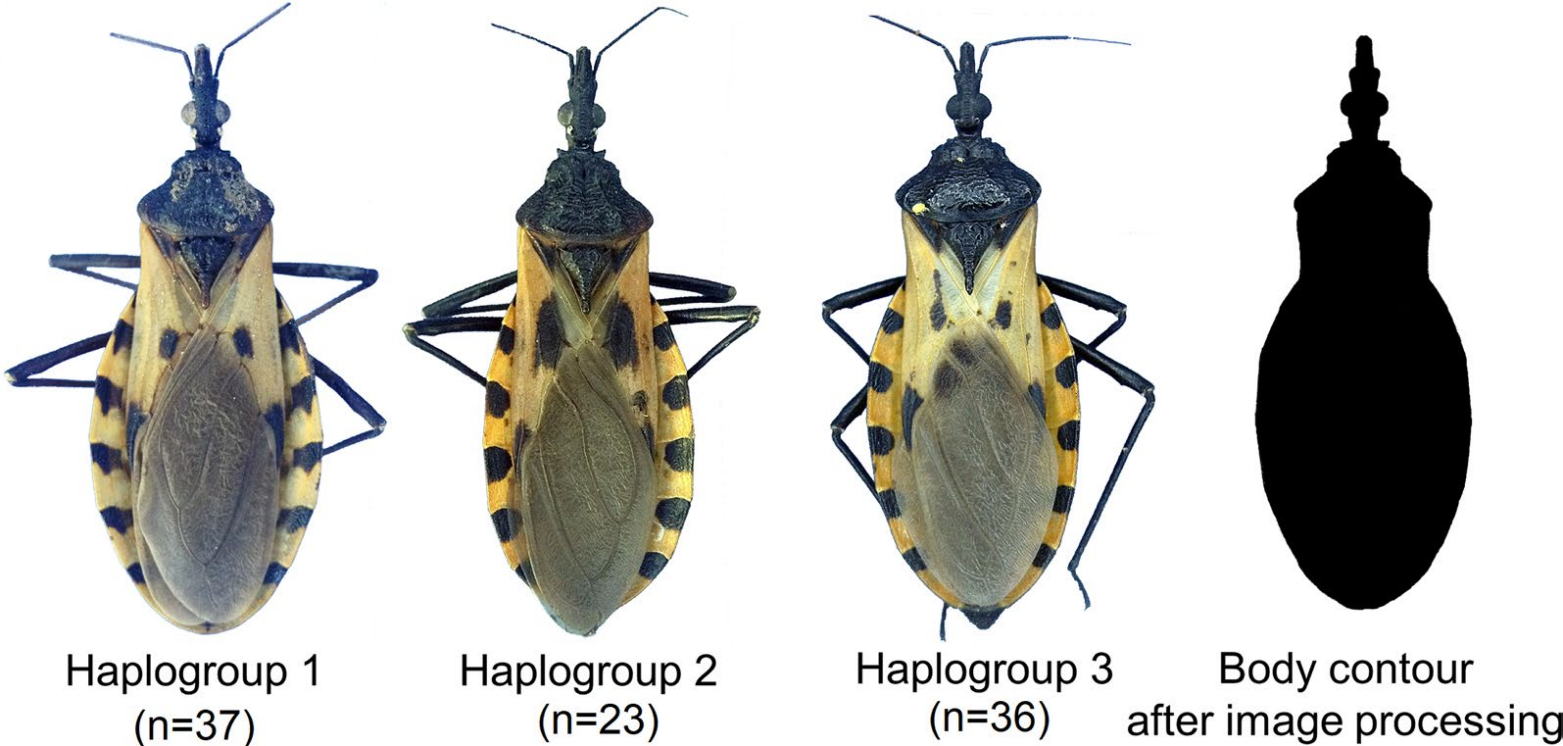
Image examples of the three haplogroups of *Triatoma dimidiata* (Hemiptera: Reduviidae) and of a body contour after image processing for the analysis of the elliptical Fourier descriptors. Sample sizes per haplogroup are shown in parentheses under each image. Copyright: Creative Commons Attribution 1.0 Universal (CC0 1.0) Public Domain Dedication license (https://creativecommons.org/licenses/by/1.0). Images modified from Gurgel-Gonçalves R, Komp E, Campbell LP, Khalighifar A, Mellenbruch J, Mendonça VJ, et al. Automated identification of insect vectors of Chagas disease in Brazil and Mexico: the virtual vector lab. PeerJ. 2017;5:e3040 [40]

The images were manually pre-processed in Adobe Photoshop CS5. This pre-processing involved the removal of the legs and antennas from each image leaving only the body contour. The brightness and contrast values were adjusted to their minimum and maximum values, respectively, to leave only a binary image (Fig. 1). All images were saved as bitmaps (BMP) in 24-bit RGB format.

### Obtention of *Triatoma dimidiata* haplogroups body contour and measurement error

To extract and quantify body contours of the *T. dimidiata* haplogroups we used SHAPE 1.3 software [54], designed to evaluate the contour shape based on elliptical Fourier transform. The observed contour is decomposed in terms of sine and cosine curves of successive frequencies called harmonics, and each harmonic is described by four coefficients. The closed contours of simple shapes can be expressed in polar coordinates with the radius as a function of the angle from a fixed internal point, which constitutes a periodic function. In this way, all the information about the shape in the sequence of points will be reduced to a smaller number of parameters whose distribution can be studied in the morphological space with the coefficients as axes [48]. Elliptical Fourier descriptors are an extension of this method, applicable when the contours are so complex that there could be more than one radius value per angle [55]. The method is developed by taking increments in X and Y between points, to define the periodic function [44]. A more detailed mathematical description of contour extraction based on EFDs can be found in Iwata et al. [56].

SHAPE has four subprograms (ChainCoder, Chc2Nef, PrinComp and PrinPrint) which together facilitate the processing of digital images, acquisition of the chain code and Fourier coefficients, and principal components analysis. It also includes routines for the visualization of the shape from previously digitized data (ChcViewer and NefViewer).

The chain code is a coding system to describe the spatial information of the contours with numbers from 0 to 7 [57]; digits indicate the direction of the next step around an outline: 0, one step to the right; 2, one step up; 4, one to the left; 6, one down; and the other digits are intermediate addresses. In order to obtain this code for each image, the ChainCoder subprogram was implemented for images of the haplogroups. This subprogram reads the BMP images, converts them to grayscale, binarizes them from a threshold value selected in the image histogram, eliminates possible noise existing in the images using erosion-dilution filters and obtains the chain code by edge detection and the contour information is stored as chain code, which is saved in an ASCII file with an extension chc. In all cases, digitization starts from the same homologous point from one image to another. Here, all images were converted to grayscale using the red channel, binarized with a threshold value of 150 and the erosion and dilution filters were worked with values of 1 and 10 respectively.

Once the chain code file was generated, for each image using the Chc2Nef program the Fourier transform coefficients for 5, 10, 15, 20, 25 and 30 harmonics were calculated consecutively, to evaluate the minimum number of harmonics that allow to achieve the best discrimination between haplogroups. We used the first harmonic ellipse parameters, to normalize the elliptic Fourier (NEF) coefficients so that they are invariant to size, rotation, and the starting position of the outline trace. NEF were stored in an ASCII file of extension.nef, and the four coefficients (related to the width-on-length ratio of the outline) were used for subsequent multivariate analyses [58].

Given that many variables (NEF) are produced (four coefficients for each harmonic), a principal components analysis (PCA) was performed using the variance-covariance matrices to reduce the dimensionality and obtain new derived variables that can be analyzed statistically. This was done using the PrinComp module, as proposed by Rohlf & Archie [48], and the scores of the first five principal components (PCs) that contributed most to the total variance were used as new shape variables. The variance contribution of all principal components is reported in Additional file 1: Table S1. The PCs contain all the information for each haplogroup body shape, as demonstrated by the fact that the contours can be graphically reconstructed from these, using an inverse Fourier transform in the PrinPrint module, according to the procedure of Furuta et al. [59]. Because in some cases, several main components can recover the contour with a high degree of precision, the first three that contributed most to the total variance were used to evaluate the interspecific and intraspecific differences in the contour. The rest of the reconstructions (the overlap between haplogroups and the individual reconstruction of each haplogroup) are shown in Additional file 2: Figure S1.

To estimate the measurement error of intraspecific variations, we produced 30 replications of 15 specimens for each haplogroups [60, 61]. Each contour of an individual was imaged and edited 30 times. An ANOSIM analysis was used to partition the total of the 1st PCs for each haplogroup into within and between-individual variations. The percentage measurement error was determined by the method indicated in Yezerinac et al. [60].

### Contour shape discrimination and statistical analysis

To evaluate the ability of FEDs to discriminate among three haplogroups of *T. dimidiata*, a discriminant function analysis was performed to determine the minimum number of harmonics needed to produce the best classifications. Here we considered as the best classification the highest percentage of correct discrimination obtained for each haplogroup. For this, the PCs recovered from the PrinComp module were used. For the first five harmonics, the number of principal components was 16, while for 10, 15, 20, 25 and 30 harmonics, 30 principal components were recovered. An ordination plot of the discriminant analysis was then generated with the PC of the minimum number of harmonics that allowed the best haplogroup discrimination and the confusion matrix was obtained to estimate the classification errors.

We also compared statistically the first five principal components among the three haplogroups. This allowed us to detect if the information related to the shape of the contour contained in the PCs presented enough differences between haplogroups. Because all the data were not normally distributed, we performed a Kruskal-Wallis test to compare among the three haplogroups.

As an alternative method of discrimination and identification, a multilayer perceptron neural networks were trained. Artificial neural networks are mathematical models constructed by simulating the functioning of biological neural networks (the nervous system). They present a set of processing units called neurons, cells or nodes (formed by several mathematical equations), interconnected by connections that include a weight that modifies the values that pass through them between neurons [61]. Artificial neural networks (ANNs) have been advocated in many disciplines for addressing complex pattern-recognition problems. The advantages of ANNs over traditional, linear approaches include their ability to model non-linear associations with a variety of data types (e.g. continuous, discrete) and to accommodate interactions among predictor variables without any a priori specification [62]. Neural networks are considered universal approximators of continuous functions, and as such, they exhibit flexibility for modeling non-linear relationships between variables. For example, ANNs exhibit substantially greater predictive power than traditional, linear approaches when modeling non-linear data (based on empirical and simulated data) [63].

The variables used to make the network were the scores of the principal components that contributed most to the total variance, obtained from the Fourier coefficients from 25 harmonics. For the basic topology, the automated search procedure of Statistica version 8.0 software was used, with an input layer of 30 neurons, corresponding to each shape variable, and the output layer with three neurons, one for each haplogroup to identify.

In the exploratory step, the most efficient network was evaluated by testing with hidden layers of between 10 and 40 neurons. Two error functions (sum of squares and cross-entropy) and four activation functions (identity, logistics, tangent and exponential) were used. The learning rate was 0.1, the inertia 0.66, and the stopping rule was set when the training error was below 0.001. Network learning was represented using the behavior of the maximum, average and minimum errors. Sixty percent of the data were randomly selected for network training and the remaining 40% was used for validation. Of the 30 networks, the one with the lowest classification error of the validation data was selected as best. The classification power for the species was analyzed using the confusion matrix and the calculation of the percentages of omission and commission errors.

## Results

### Measurement error of intraspecific variations and statistical difference in shape

On the first PC (the one that most contributed to the total variance), the percentage measurement error reached 2.3% of the intraspecific variance for the haplogroup 1, 3.3% for the haplogroup 2 and 3.6% for the haplogroup 3.

### Contour reconstruction and variance explained by PCA

**Fig. 2:**
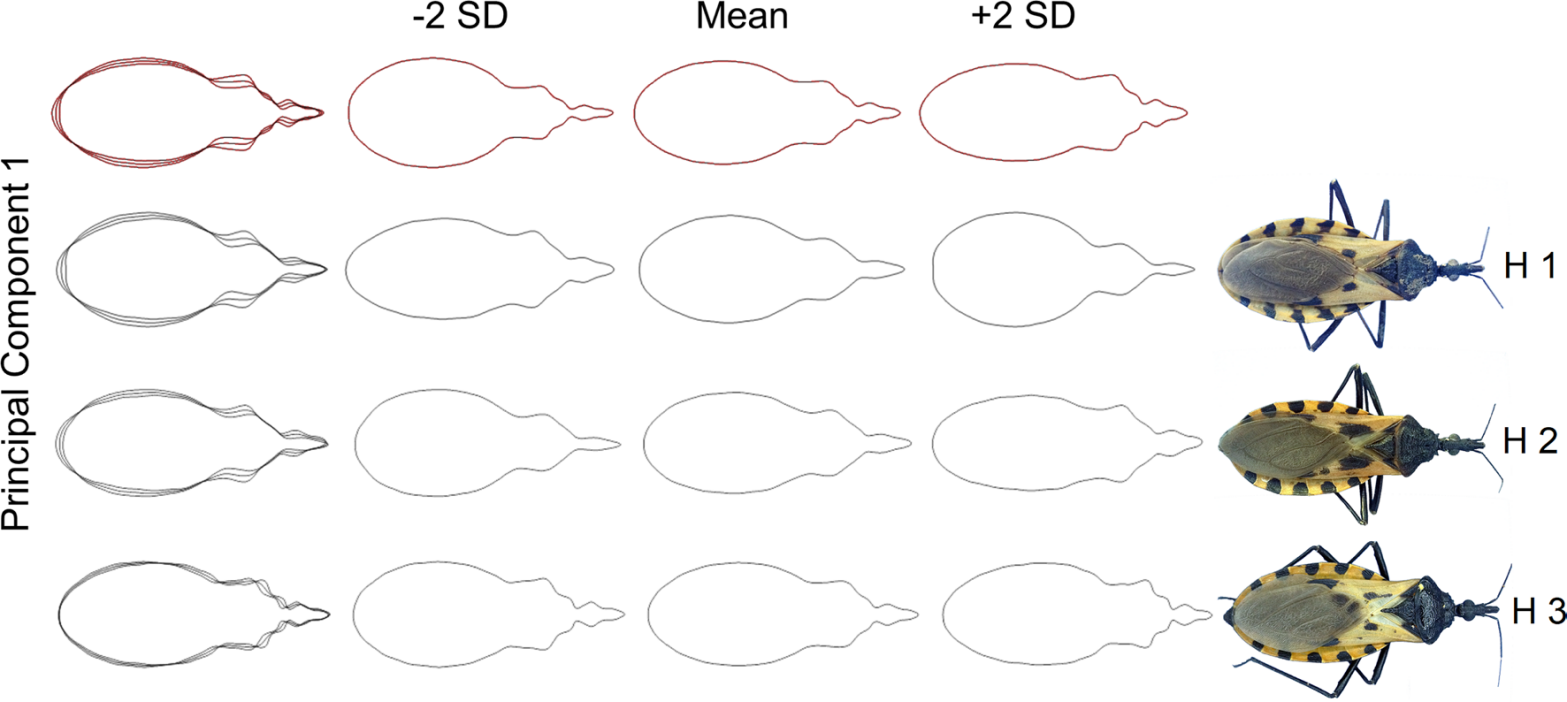
Digital reconstruction and variability of the contours in three haplogroups of *Triatoma dimidiata* (Hemiptera: Reduviidae). The contours were obtained from the first principal components obtained with the elliptical Fourier descriptors. Red contours represent the consensus of the three haplogroups. *Abbreviations*: H1, haplogroup 1; H2, haplogroup 3; H3, haplogroup 3

With the result of the first component, and when using the inversion of the Fourier transforms, the contour of the haplogroups of *T. dimidiata* was reconstructed and the variability among and within groups was graphically characterized (Fig. 2). The greatest variability among haplogroups was observed in the posterior lobe of the pronotum and the terminal region of the head and neck. This pattern of variation was also observed internally within haplogroups 1 and 2. The greatest variation within haplogroup 3 specimens was in the anterior lobe and distal tubers (Fig. 2).

**Fig. 3:**
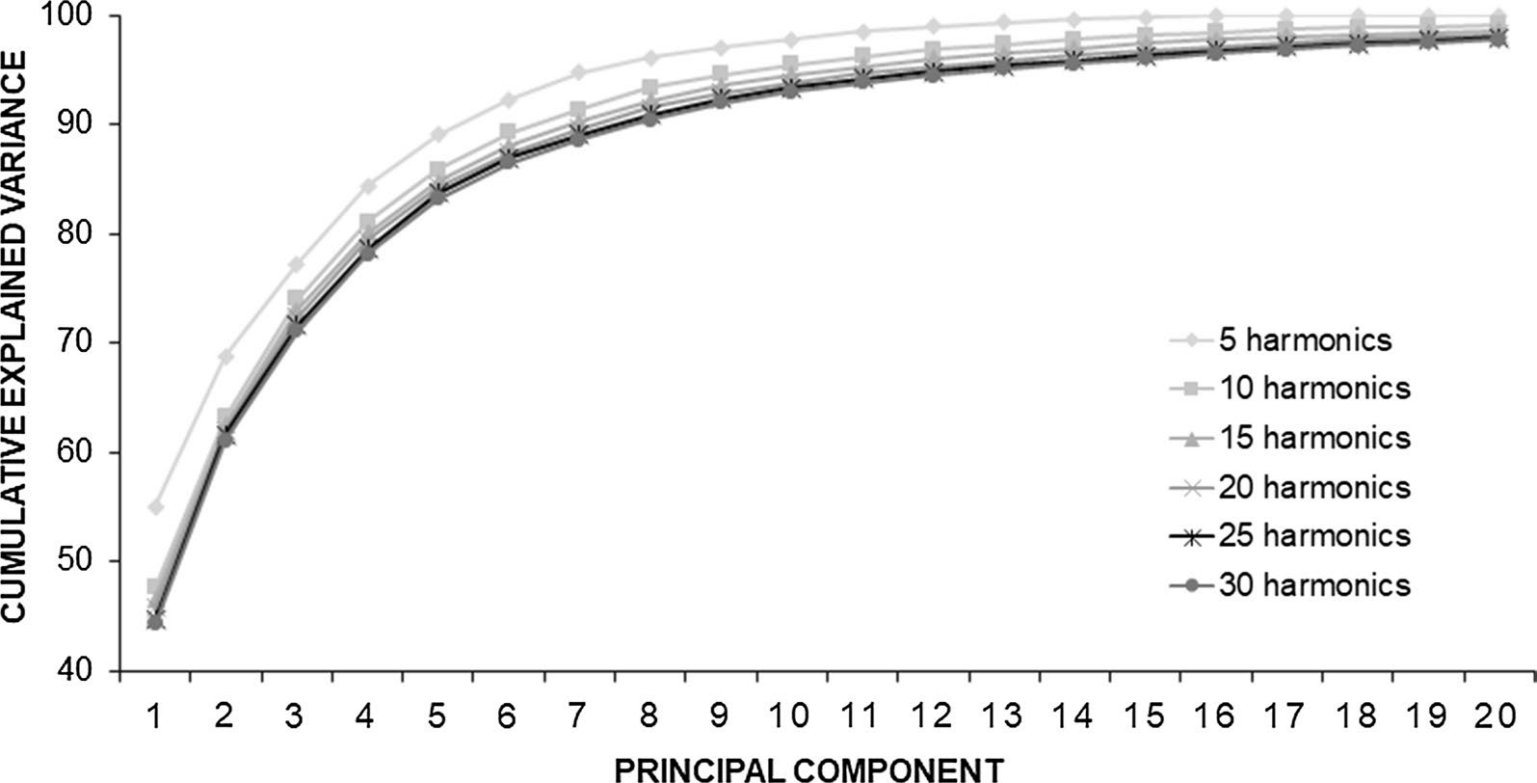
Behaviour of the variance explained by the principal components as the number of harmonics used to characterize the shape of three haplogroups of *Triatoma dimidiata* (Hemiptera: Reduviidae) is increased

Regardless of the number of harmonics used to describe the contour, the first component explained about half of the contour variability (between 44–55%) (Fig. 3). As the number of harmonics used increased, more components were required to explain 90% of the variation in shape, but in general, this value was reached with 8 principal components (Fig. 3).

### Discriminant analysis and neural network

When performing the discriminant analysis to assess the number of harmonics that offers the best discriminations among haplogroups, it was observed that correct discriminations generally increased with the number of harmonics used. This pattern stopped at 30 harmonics when correct discrimination began to fail (Table 1). Haplogroup 1 was successfully differentiated 10 harmonics with 100% correct discrimination. Haplogroup 2 reached a 100% correct discrimination when the contour was described with 20 and 25 harmonics. Haplogroup 3 only reached 88.24% and 94.12% correct discrimination with the maximum number of harmonics tested. Overall, the best results were obtained when describing the contours of haplogroups using 25 harmonics.

**Table 1:** Percentage of correct discrimination for three haplogroups of *Triatoma dimidiata* (Hemiptera: Reduviidae) for discriminant analysis using 5, 10, 15, 20, 25 and 30 harmonics

No. of harmonics	Correct classification (%)			Total
	Haplogroup 1	Haplogroup 2	Haplogroup 3	
5	97.14	60	88.24	85.39
10	100	85	94.12	94.38
15	100	90	94.12	95.51
20	100	100	91.18	96.63
**25**	**100**	**100**	**94.12**	**97.75**
30	100	95	94.12	96.63

*Note*: The best correct discrimination values for a certain number of harmonics are highlighted in bold

**Fig. 4:**
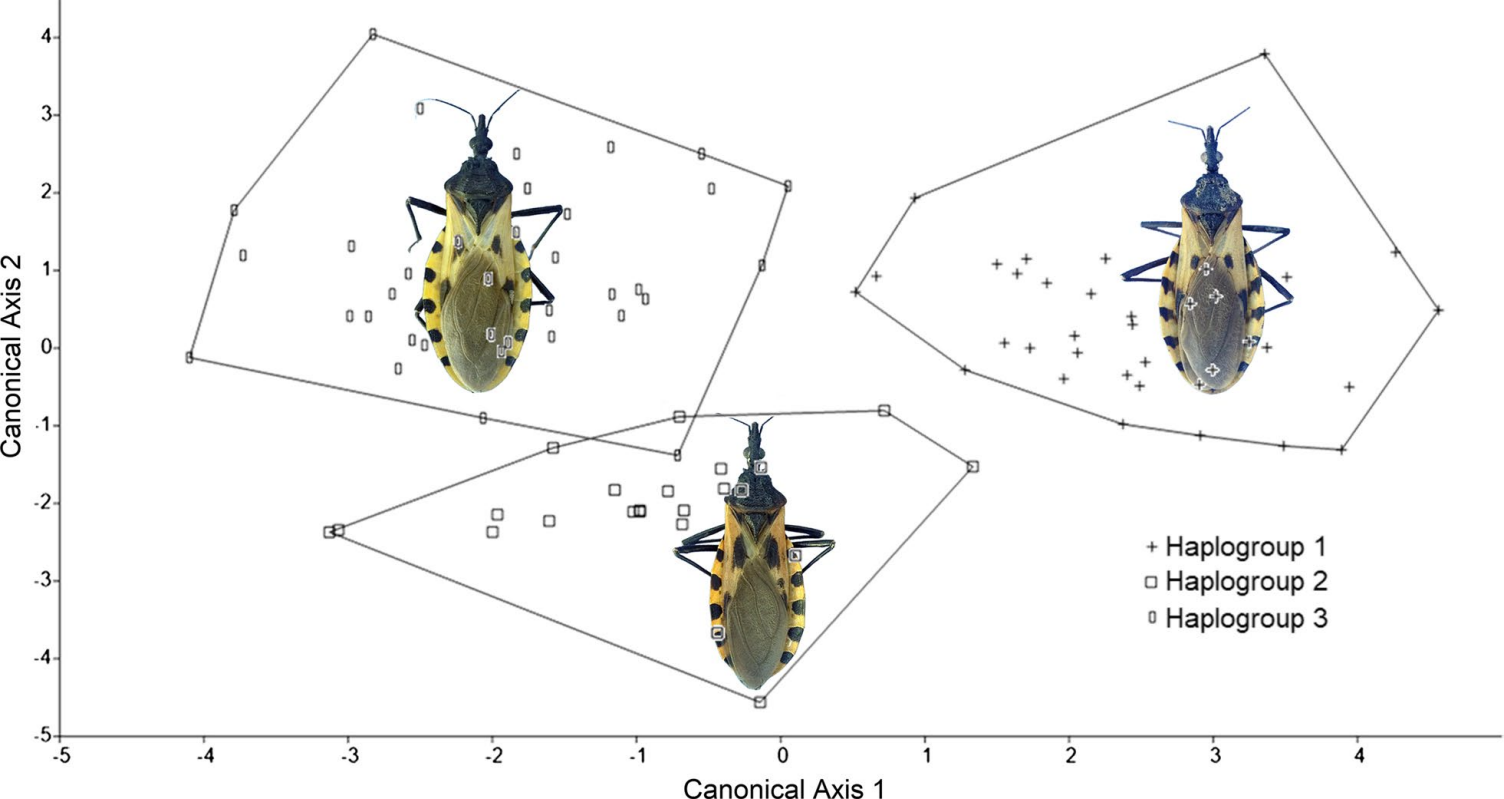
Ordination plot of the discriminant analysis using shape variables 30 principal components resulting from the elliptical Fourier coefficients of 25 harmonics of three haplogroups of *Triatoma dimidiata* (Hemiptera: Reduviidae)

The ordering diagram of the discriminating axes for the shape of the specimens, for the description of the contour with 25 harmonics and using 30 PCs is shown in Fig. 4. The separation of the minimum convex polygons demonstrated the possibility of discriminating the haplogroups using the PC as shape variables. Haplogroup 1 is separated perfectly from the rest, showing the greatest differentiation from haplogroups 2 and 3 along canonical axis 1. Haplogroups 2 and 3 presented greater variation along canonical axis 2. One individual from haplogroup 3 was located within the polygon of haplogroup 2, which was corroborated as an error of discrimination of the analysis in the confusion matrix (Table 2).

**Table 2:** Confusion matrix of the discrimination process of the three haplogroups of *Triatoma dimidiata* (Hemiptera: Reduviidae) for the ordination plot of the discriminant analysis for 25 harmonics

Haplogroup	H1	H2	H3	Total
H1	37	0	0	37
H2	0	23	0	23
H3	0	1	35	36

**Fig. 5:**
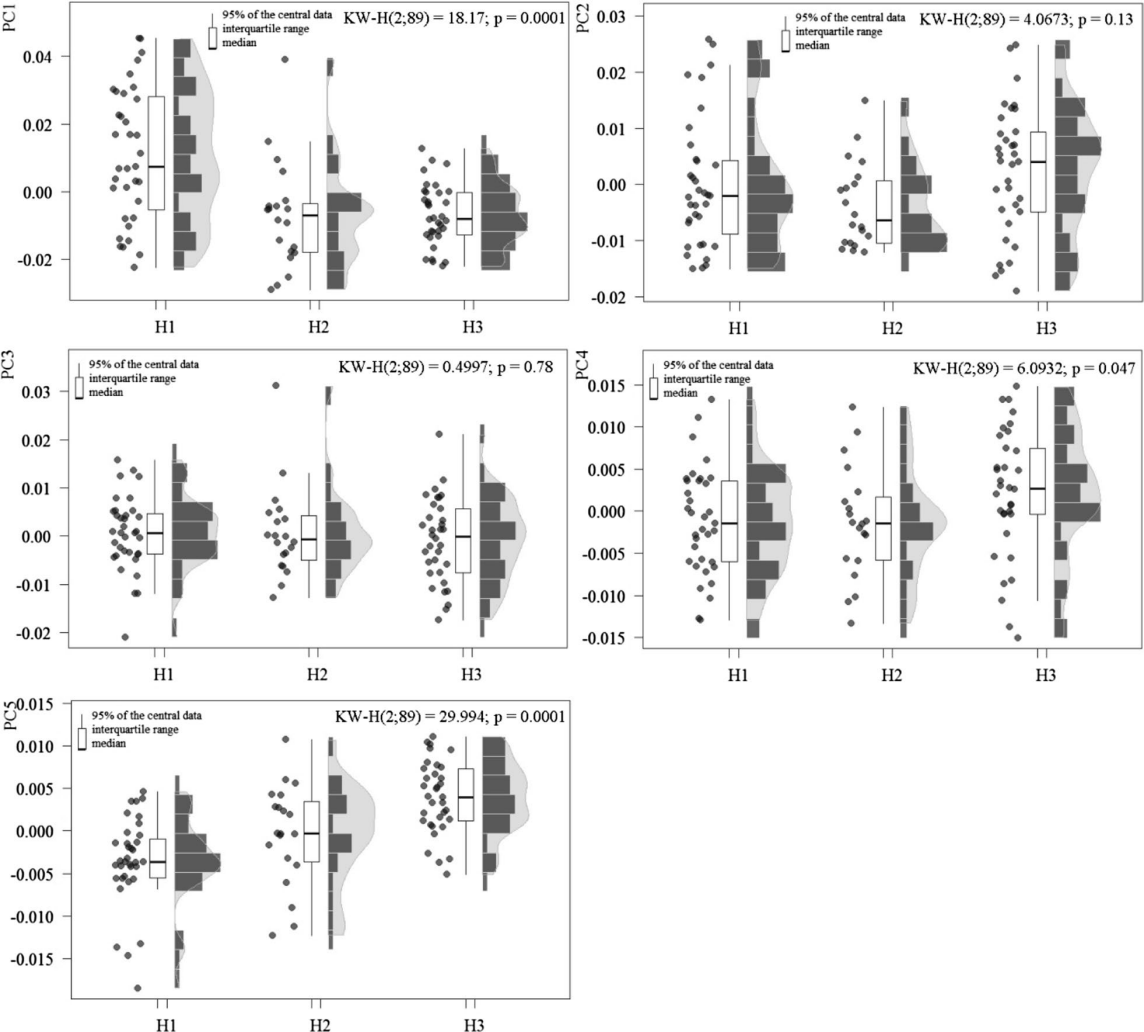
Score differences in the first five principal components among three haplogroups of *Triatoma dimidiata* (Hemiptera: Reduviidae). *Abbreviations*: H1, haplogroup 1; H2, haplogroup 2; H3, haplogroup 3

When comparing the first five PC scores among the three haplogroups, significant differences were found among, at least, two haplogroups for all principal components except for PC2 and PC3 (Fig. 5). The greatest differences were always found between haplogroups 1 and 3.

All trained networks achieved 100% correct classification with training data, but the most efficient with validation data was a perceptron of 13 neurons in the hidden layer, which reached 91% correct classification. This network used a BFGS18 training algorithm and an SOS error function. The activation function of the hidden layer was ‘Logistics’ and for the output layer, ‘Tangent’. This network confused only one individual of haplogroup 1 (out of a total of 20) which was classified as haplogroup 2 (dropping to 94% of correct classification), and two individuals of haplogroup 2 (of 16) were classified as haplogroup 3 (for 85% of correct classification). The eight individuals belonging to haplogroup 2 used to validate the network were correctly classified (100%). Components 1, 5, 15 and 2 had the highest weight in the network.

## Discussion

In entomological studies, much attention has been given to the use of different parts of the body to identify, name and classify insects [64]. To date, wings have been the most commonly used structures to assess species discrimination through geometric morphometry methods, mainly using anatomical reference points [7, 26]. Here, to the best of our knowledge, we use for the first time the whole-body contour of an insect to discriminate among haplogroups. The use of EFDs has been little explored, though on several occasions it has demonstrated its ability to discriminate among even closely related species [65, 66]. Even more, some studies with triatomines [27, 53] and other insects of medical importance [27, 67–69] have demonstrated the usefulness of these methods for species recognition in this genus.

When using the inversion of the Fourier transforms, it was possible to visualize that the greatest differences in contour shape between the haplogroups were found in the pronotum and the head. Both structures have been used in morphometric studies, both traditional and geometric, because important variations in their shape have been detected [7, 14, 40]. In the case of the head, Bustamante et al. [14] consider that an important factor in the variability observed in this region is due to the geographical isolation of the populations of *T. dimidiata*, which has led to divergent evolution. The haplogroups used in this study mainly have allopatric populations, which could explain the morphometric differences found, although there are areas of sympatry [18]. In turn, differences in the head may have an evolutionary cause related to feeding strategies and growth patterns of this area of the body. Some authors have suggested that the shape of the head may reflect evolutionary mechanisms related to the ability to ingest blood. If the allopatry of the haplogroups populations of *T. dimidiata* is taken into account and that these must have diverged approximately 0.97 to 0.85 mya, according to results obtained from sequences of the *nad*4 gene [16], dissimilar feeding strategies may have been established among the haplogroups, which then generated morphological differences. However, because the information related to the localities where individuals where obtained is not available in the original paper [40], more precise conclusions cannot be reached.

In the case of the pronotum, significant variability in the shape of the contour between the haplogroups was also observed. This structure has been used in the traditional morphological description of triatomine species [9] and has been used in attempts to discriminate species [50]. To our knowledge, only one study has used geometric morphometry techniques on this structure in triatomines [32], in the future a comparative study of the pronotum could evaluate its utility in the discrimination among triatomines.

When comparing the results obtained by Gurgel-Gonçalves et al. [40], who reached correct discrimination values between 70.5% and 82.5% of the three haplogroups and the results obtained by Khalighifar et al. [41] (with correct discrimination values of 84.1% H1, 86.7% H2 and 87.5% H3) our results reached 100% correct discrimination values for haplogroup 1 and 2 and 94.12% for haplogroup 3, with total discrimination results of 97.75%, through discriminant function analysis. This is probably because, in comparison to the methods used in the studies mentioned above, EFDs can recover a greater variability of the shape through the contour analysis. Perhaps the integration of both, the methods of the previous studies [40, 41] (which have demonstrated their ability with good values of correct discrimination in the recognition of these haplogroups) and EFDs, can help to establish an identification system of the haplogroups of *T. dimidiata* with higher values of correct identification.

This method of describing shapes and reconstructing images is advantageous when the analysis based on anatomical reference points fails to fully discriminate the objects of study. McLellan and Endler [70] suggested that the use of EFDs provides a precise reconstruction of the contour of the complex object and can explain the overall complexity of the shape with greater resolution than the methods of anatomical reference points and semi-landmarks. This has been demonstrated in other insect groups, where the use of EFDs has allowed the correct discrimination between species [71]. Francoy et al. [24] used both methods (anatomical reference points and elliptical Fourier descriptors) for the identification of euglossine bees. These authors found better results in the differentiation of species using EFD. However, they suggest the combined use of data matrices obtained by anatomical reference points and EFDs.

Species concepts and delimitation have always been highly controversial and complicated, especially when the focal organisms are considered cryptic or hypercryptic [64]. In the *Triatoma* genus, the presence of cryptic species has been widely addressed. Several authors have assembled the *Triatoma* species into different groups and complexes based on their external characters and the genitalia of both sexes [72–74]. Currently, the most accepted group was proposed by Schofield and Galvão [74], with the subdivision of *Triatoma* species into groups, complexes and subcomplexes.

Triatominae species show high morphological variation, which suggests that ecological factors may be the main force driving speciation in the Triatominae [22]. Very closely related species can develop rapid morphological changes in adaptation to new environments. Conversely, similar morphs adapted to the same ecotope could be derived from different ancestors [22]. Thus, the existence of morphologically similar species could be reflecting their evolution from a common ancestor or convergent adaptation to the same ecological niche. This phenotypic flexibility leads to the misidentification of distinct genetic units by morphological convergence, resulting in taxonomic uncertainties in the description of new subspecies, species or even genera. Considering that the Triatominae species groupings into complexes and subcomplexes are mainly based on morphological similarities [75], the morphological plasticity complicates both species identification and the establishment of evolutionarily related groups. In this sense, traditional morphological analysis has failed to clarify the differences that other sources of evidence, such as genetic, chromosomal, karyotype analyses, etc., have contributed to the clarification of the cryptic species complexes.

Specifically, in *T. dimidiata*, wide distribution and variation in morphology (historically explained by wide clinal variation along its distribution range) [11], has resulted in a long history of reconsiderations of its taxonomic status, from a single species to a species complex of distinct taxonomic groups [76]. Studies focused on the analysis of morphological variation using classical morphometry techniques have led to the inclusion of *T. dimidiata* populations within other species complexes such as *phyllosoma* [14]. However, these considerations have been rejected due to genetic evidence that has demonstrated the presence of different haplogroups within the *dimidiata* complex; this demonstrates that it is impossible for classical morphological techniques to correctly discriminate among these haplogroups.

## Conclusions

The use of elliptic Fourier descriptors allows the identification of three haplogroups of *Triatoma dimidiata* with higher precision than previous works, where higher values of correct discriminations were 82.5% [40] and 87.5% [41]. With 25 harmonics and 30 components, the identification of haplogroups was achieved with an overall efficiency greater than 97% by using discriminant analysis. The multilayer perceptron neural networks were also efficient in identification, reaching 91% efficiency with the validation data. The main advantage is its easy application from easily obtainable digital images with minimal and uncomplicated processing, which guarantees its replicability. Despite its relative mathematical complexity, it can be partially automated, which minimizes the researcher manipulation errors when processing the samples. Its ability to reconstruct the shape automatically, after statistical processing, is also attractive and does not require any drawing skills from the researcher, allowing the visual identification of the location of the differences detected. The assessment of the identification ability of this method in other triatomine species is a necessary aspect to advance procedures that allow the automation of the identification of these important vectors of Chagas disease.

## Supplementary information

**Additional file 1: Table S1.** Variance contribution of all principal components obtained with the PrinComp module. https://doi.org/10.6084/m9.figshare.12014976.v1

**Additional file 2: Figure S1.** Digital reconstruction and variability of the contours in three haplogroups of *Triatoma dimidiata* (Hemiptera: Reduviidae), obtained from all principal components derived from the elliptical Fourier descriptors. https://doi.org/10.6084/m9.figshare.12014979.v1.

## Data Availability

Data supporting the conclusions of this article are included in the article and its additional files. Raw data are available upon request to the first author. Also, all data derived from this investigation are deposited in the Figshare repository (10.6084/m9.figshare.11344073.v1)

## Abbreviations

CD: Chagas disease
EFD: Elliptic Fourier descriptors
H1: Haplogroup 1
H2: Haplogroup 2
H3: Haplogroup 3
