# Supplementary material for: Identifying Chagas disease vectors using elliptic Fourier descriptors of body contour: a case for the cryptic dimidiata complex
Source: Parasit Vectors. 2020 Jul 1;13:332. doi: 10.1186/s13071-020-04202-2 (PMC7329423; doi:10.1186/s13071-020-04202-2)
Supplement: Supplementary file 2 — Additional file 2: Figure S1. Digital reconstruction and variability of the contours in three haplogroups of Triatoma dimidiata (Hemiptera: Reduviidae), obtained from all principal components derived from the elliptical Fourier descriptors. https://doi.org/10.6084/m9.figshare.12014979.v1. [file 13071_2020_4202_MOESM2_ESM.pdf]

Additional file 2: Figure S1. Digital reconstruction and variability of the contours in three haplogroups of *Triatoma dimidiata* (Hemiptera: Reduviidae), obtained from all principal components derived from the elliptical Fourier descriptors.

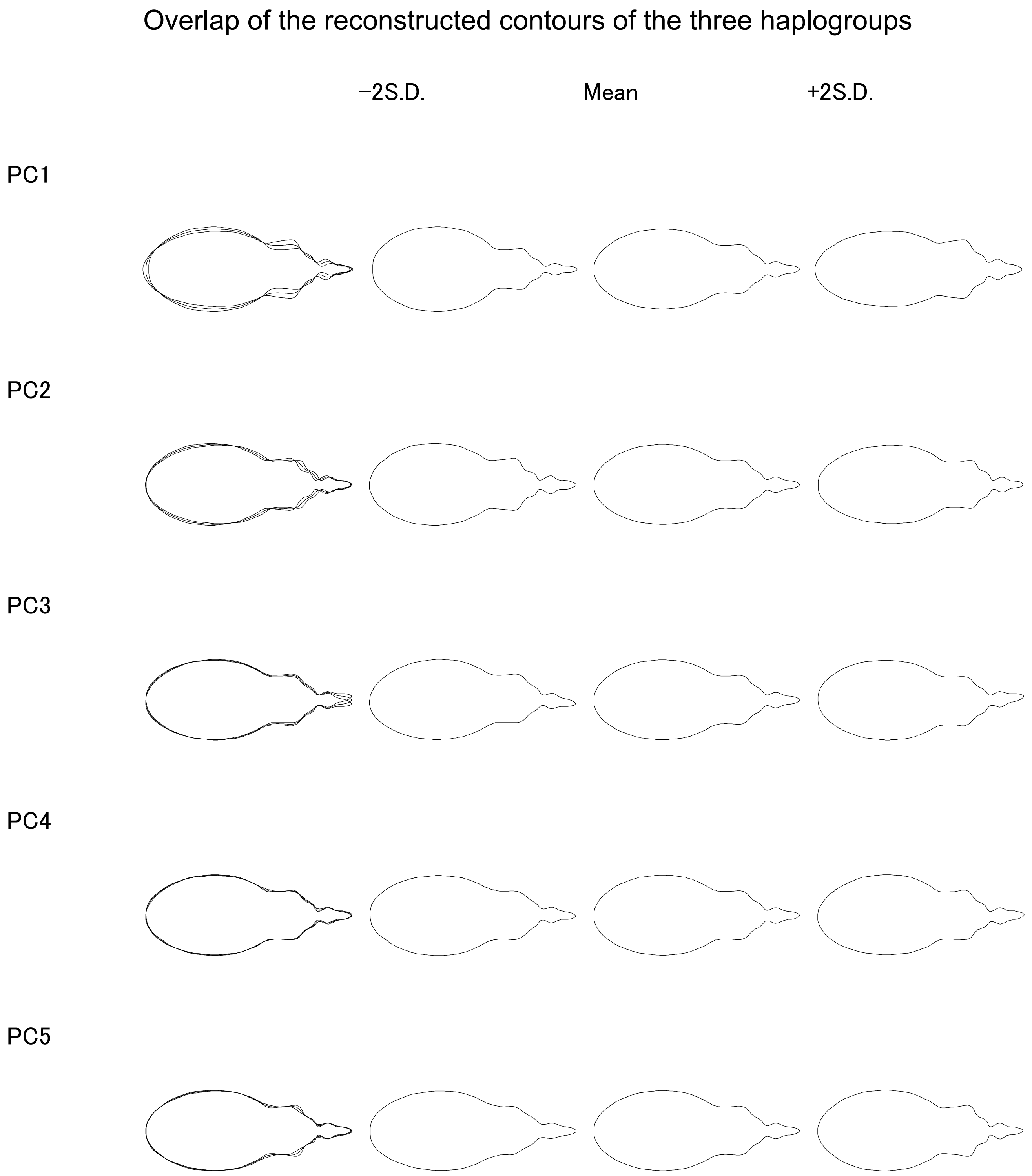

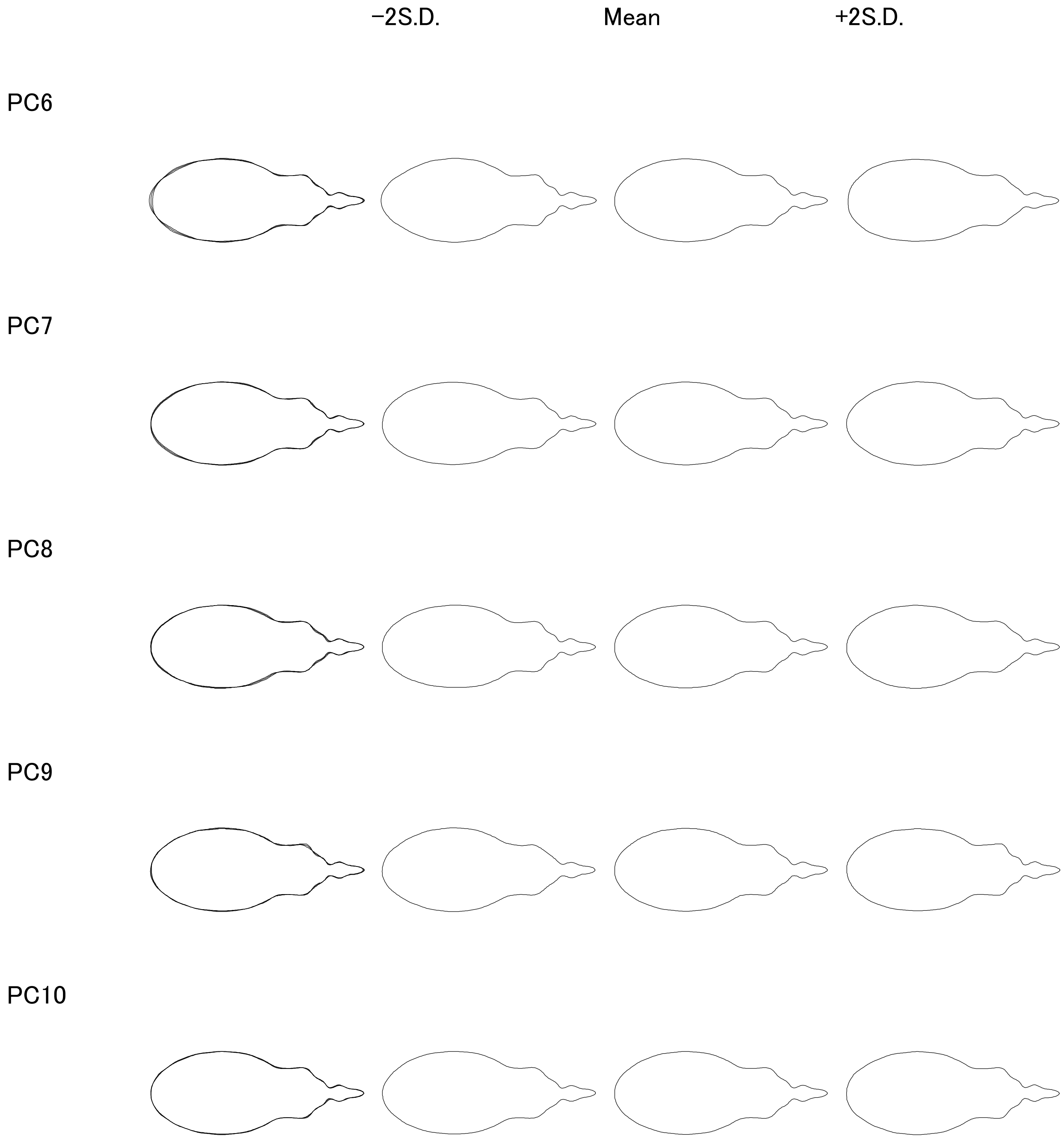

−2S.D.
Mean
+2S.D.
PC6
PC7
PC8
PC9
PC10

Reconstructed contour of the haplogroup 1

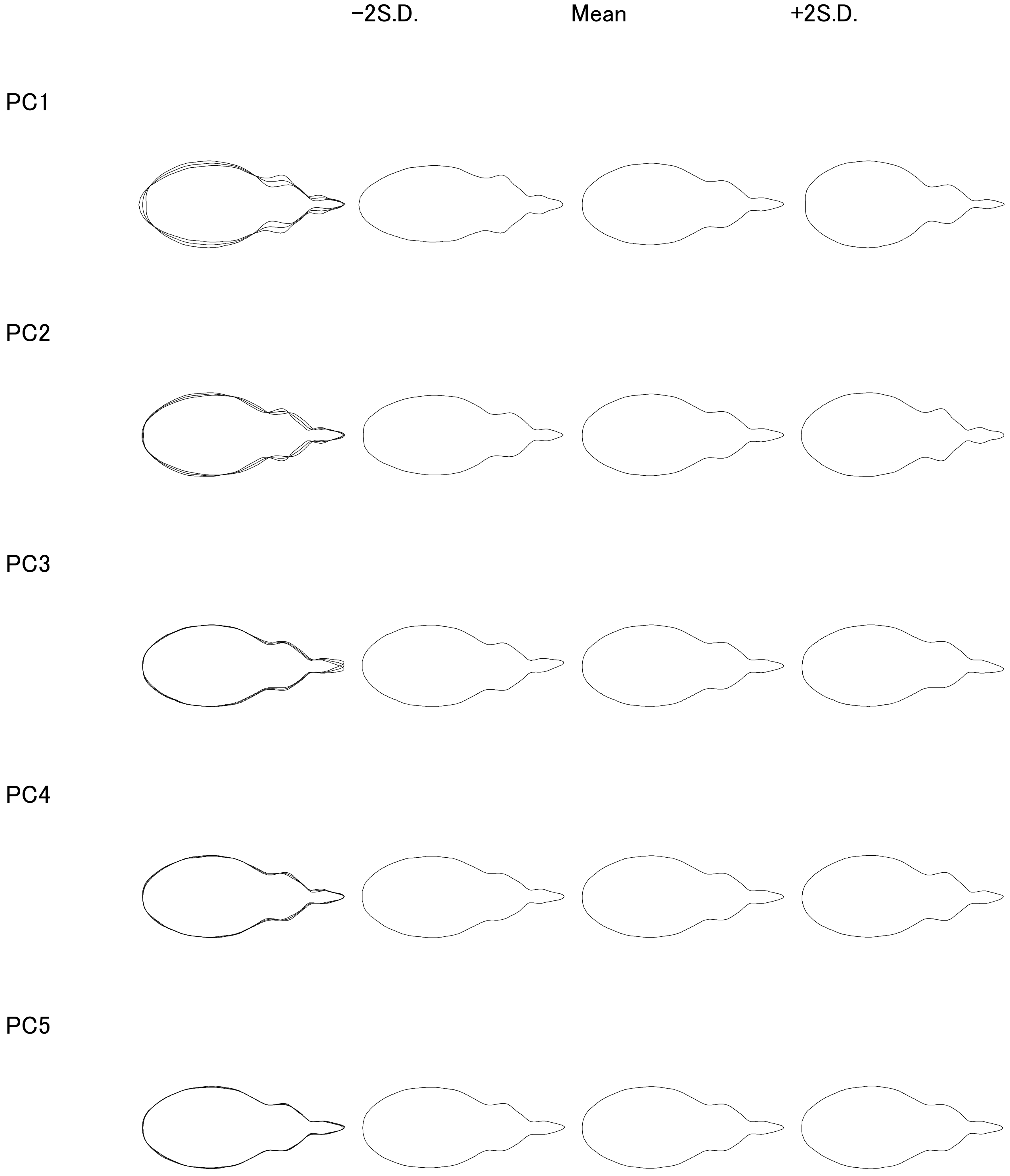

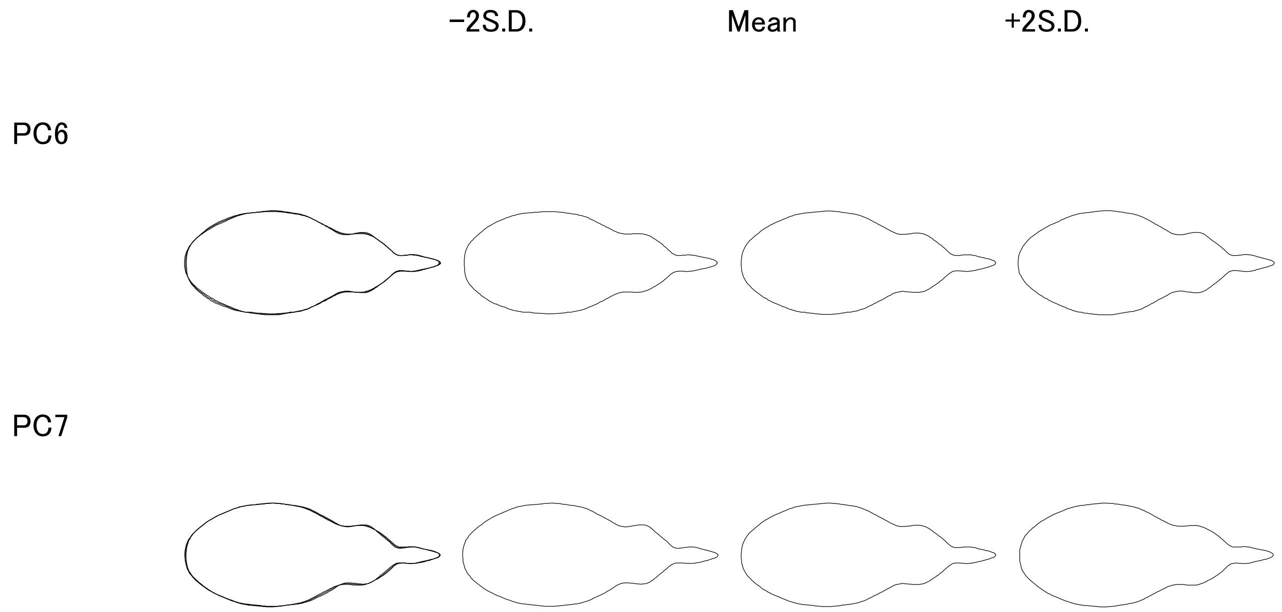

−2S.D.
Mean
+2S.D.
PC6
PC7

Reconstructed contour of the haplogroup 2

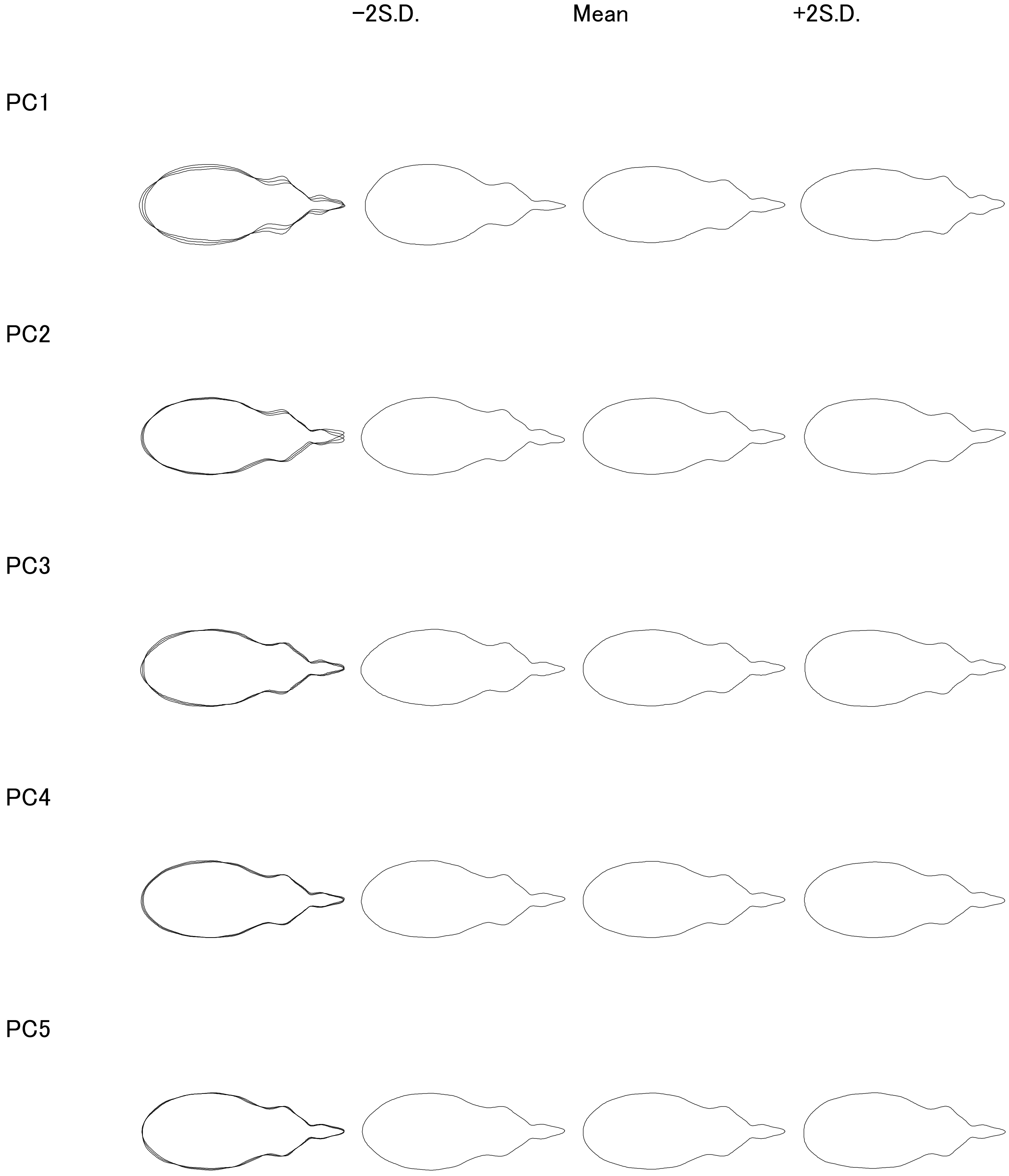

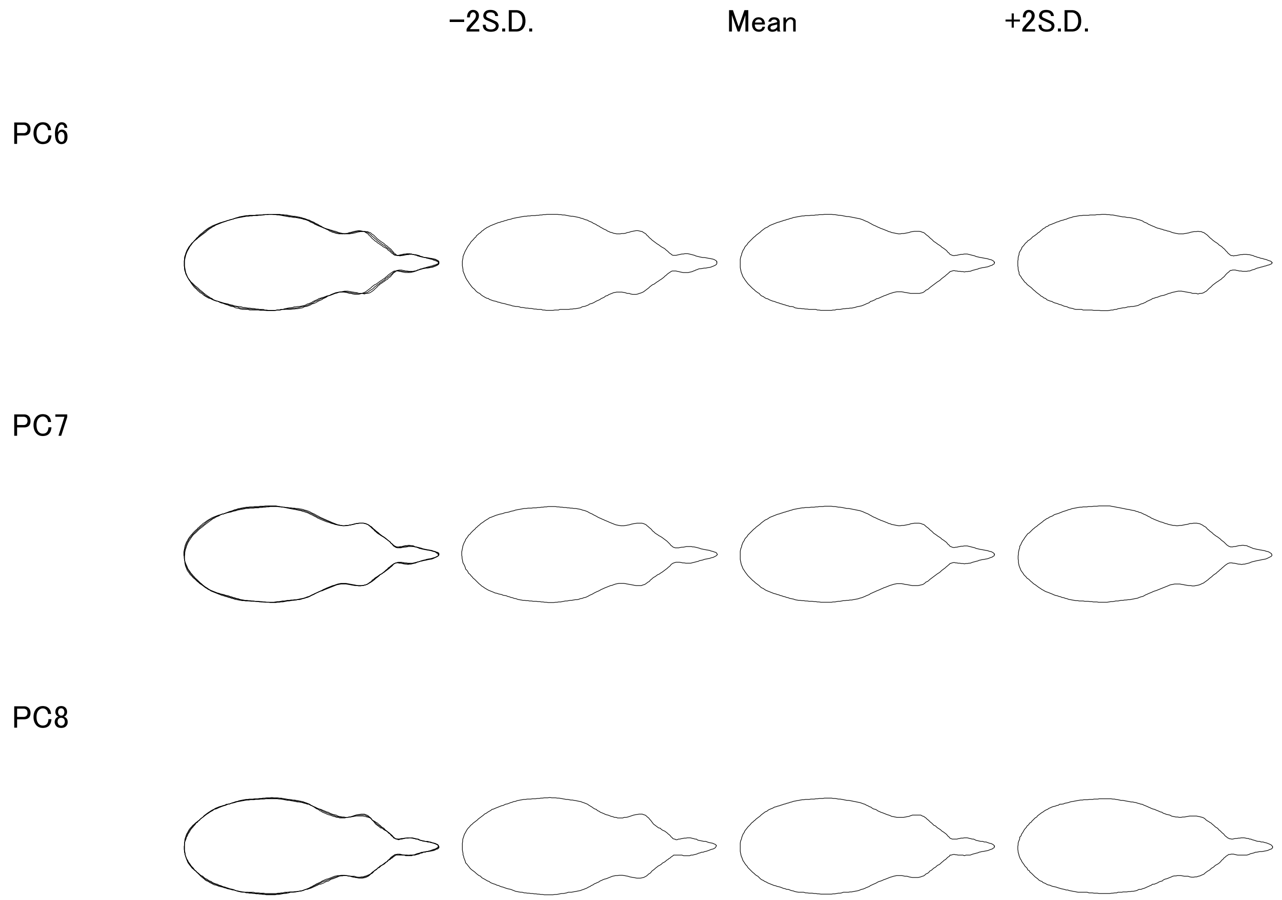
−2S.D.
Mean
+2S.D.
PC6
PC7
PC8

Reconstructed contour of the haplogroup 3

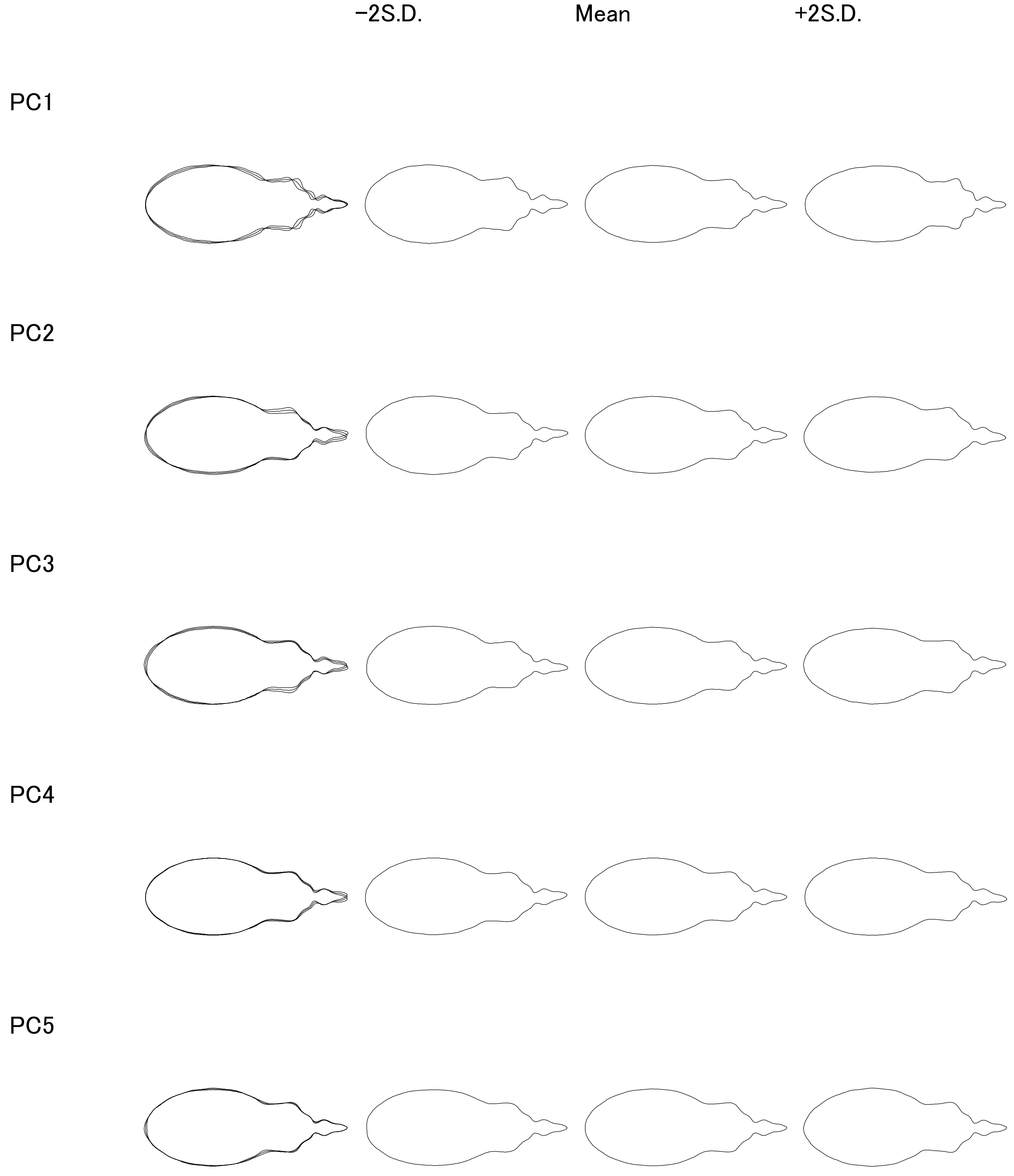

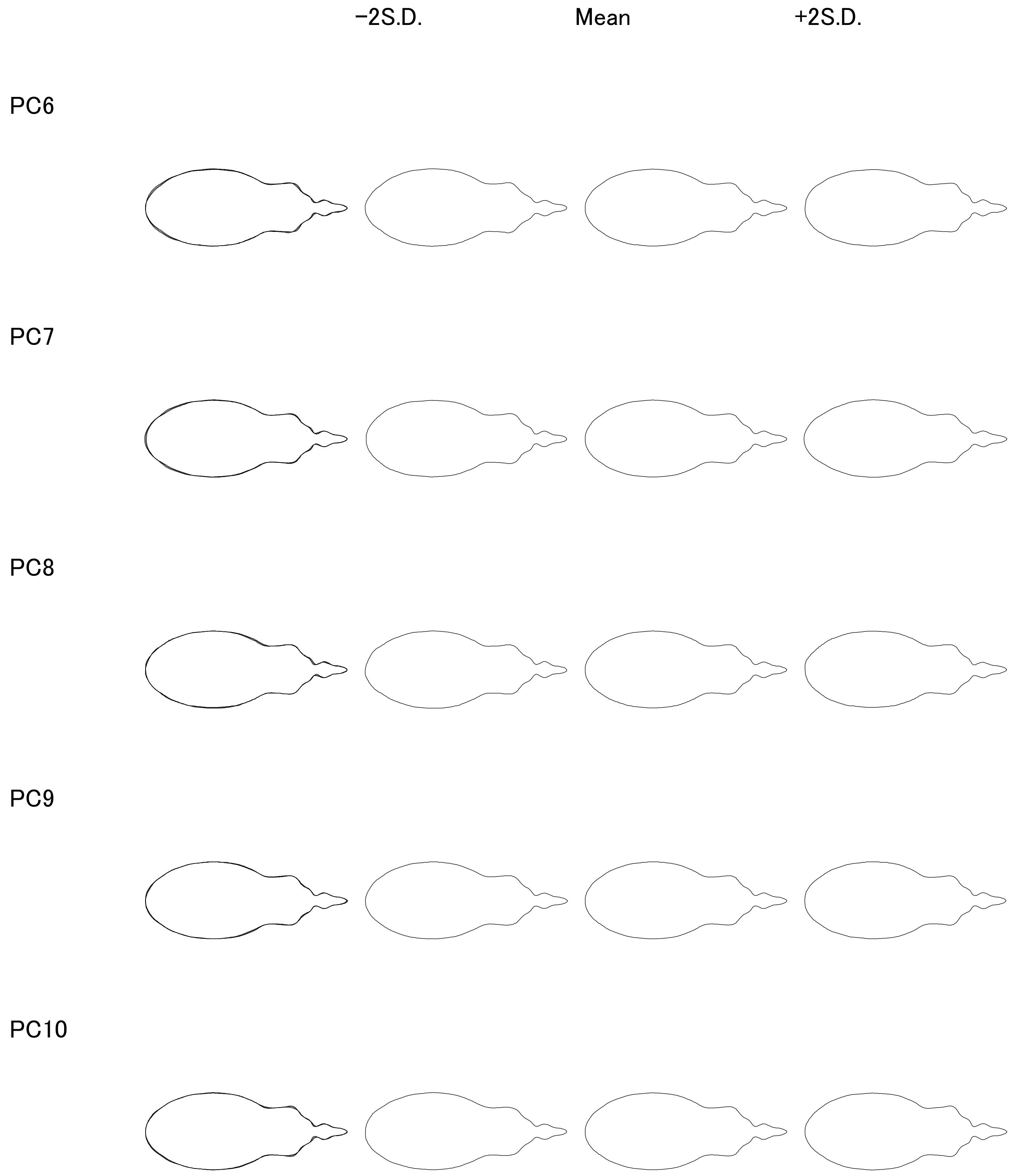

−2S.D.
Mean
+2S.D.
PC6
PC7
PC8
PC9
PC10

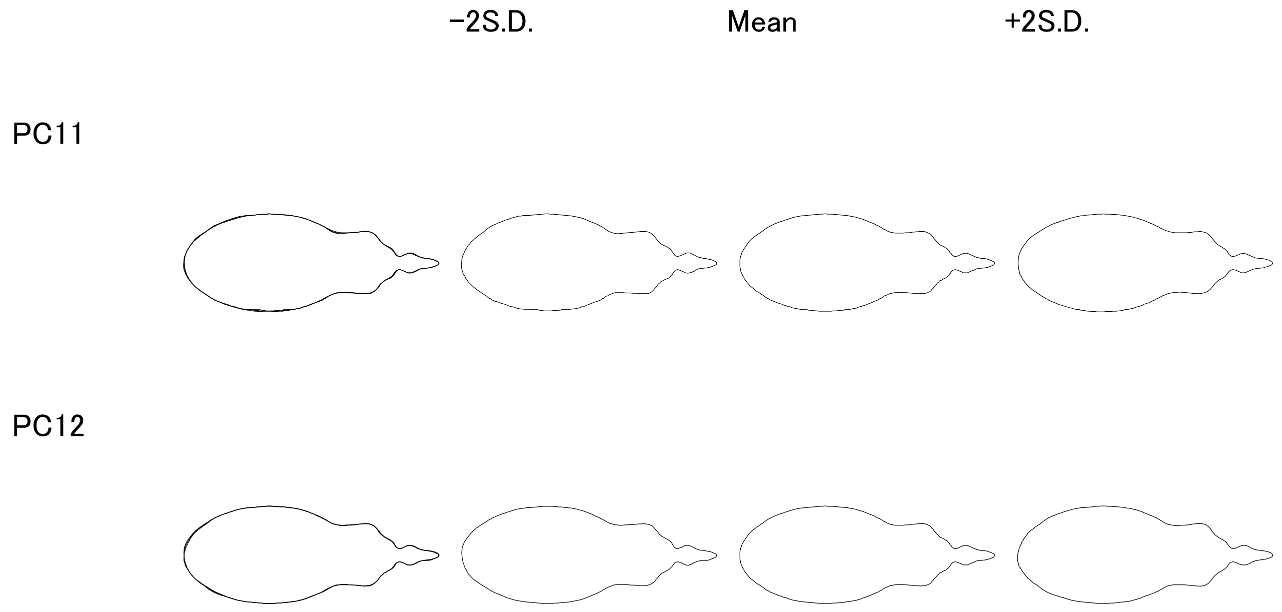
−2S.D.
Mean
+2S.D.
PC11
PC12
